# Is more involvement needed in the clinical trial design & endpoints?

**DOI:** 10.1186/1750-1172-7-S2-A38

**Published:** 2012-11-22

**Authors:** Elizabeth Vroom

**Affiliations:** 1 Duchenne Parent Project, Amsterdam, 1017JG The Netherlands

## 

Duchenne muscular dystrophy (DMD) is a recessive X-linked form of muscular dystrophy, affecting around 1 in 3,600 boys, which results in muscle degeneration and eventual death. Long before any promising drug was at the horizon Duchenne parents came forward to organise research meetings where they made it clear they were willing to shoulder responsibility and contribute towards advancing treatments and a cure. They became funders of peer reviewed research and advocated for government support. Some organisations started their own research institutes others invested in extramural research, clinical centers and industry to develop viable treatments for DMD and BMD. Currently several potential drugs are in phase 3 trials.

Through this experience Duchenne parents have learned including Patient Organisations in trial design and selection of endpoints can make drug development more efficient. Patients participate in clinical trials and will ultimately be the ones to decide whether the tested drugs are beneficial to them to an extent that EMA and FDA allow these drugs. Hence patient input and advice is essential to develop a clinically meaningful endpoint. A clinical meaningful endpoint is an endpoint that directly measures how a patient feels (symptoms), functions (the ability to perform activities in daily life), or survives. Therefore, a primary endpoint should be a direct measure of one of these. A primary endpoint should generally not be a measure of something that is not important to the patient [1]. Who knows better than the patients what is important to them? It is remarkable that clinicians and industry often don’t discuss the choice of primary outcome measures with patient organisations in an early phase, but only after they have already collected the data and are at the point to discuss outcomes with regulators.

Regarding trial design, ethic committees mostly decide about the burden and risk of participation in relation to a certain trial design without asking the patients or patients’ organisations for their opinion. That is remarkable because they are the ones who have to deal with the burden and take the risk. Often researchers and regulators only look at the burden of the medical intervention, where for many patients other factors add much more to the total burden of participation in a trial.

Care revolves around the people with a disease. Without them there would be no need for research and drug development. It is important that the needs of these groups are the starting point for initiatives concerning them. Patients know what it means to have this condition. It means they will bring in a different perspective. Their questions and needs are based on their own experiences, interests and vision. There is a lot to gain from well utilised experience and expertise.
